# Present practices and emerging opportunities in bioengineering for slope stabilization in Malaysia: An overview

**DOI:** 10.7717/peerj.10477

**Published:** 2021-01-12

**Authors:** Deivaseeno Dorairaj, Normaniza Osman

**Affiliations:** Institute of Biological Sciences, Faculty of Science, University of Malaya, Kuala Lumpur, Malaysia

**Keywords:** Bioengineering, Slope stabilization, Slope failure, Current practices, Mix-culture

## Abstract

Population increase and the demand for infrastructure development such as construction of highways and road widening are intangible, leading up to mass land clearing. As flat terrains become scarce, infrastructure expansions have moved on to hilly terrains, cutting through slopes and forests. Unvegetated or bare slopes are prone to erosion due to the lack of or insufficient surface cover. The combination of exposed slope, uncontrolled slope management practices, poor slope planning and high rainfall as in Malaysia could steer towards slope failures which then results in landslides under acute situation. Moreover, due to the tropical weather, the soils undergo intense chemical weathering and leaching that elevates soil erosion and surface runoff. Mitigation measures are vital to address slope failures as they lead to economic loss and loss of lives. Since there is minimal or limited information and investigations on slope stabilization methods in Malaysia, this review deciphers into the current slope management practices such as geotextiles, brush layering, live poles, rock buttress and concrete structures. However, these methods have their drawbacks. Thus, as a way forward, we highlight the potential application of soil bioengineering methods especially on the use of whole plants. Here, we discuss the general attributions of a plant in slope stabilization including its mechanical, hydrological and hydraulic effects. Subsequently, we focus on species selection, and engineering properties of vegetation especially rooting structures and architecture. Finally, the review will dissect and assess the ecological principles for vegetation establishment with an emphasis on adopting the mix-culture approach as a slope failure mitigation measure. Nevertheless, the use of soil bioengineering is limited to low to moderate risk slopes only, while in high-risk slopes, the use of traditional engineering measure is deemed more appropriate and remain to be the solution for slope stabilization.

## Introduction

Development often involves mass land clearing, without which, it is almost impossible to cater to the needs of the urban population. However, excessive land clearing and unplanned development could pose irreversible environmental hazards such as habitat destruction, loss of biodiversity, soil erosion and environmental pollution (Lateh & Ahmad, 2011). Moreover, land scarcity has not only encroached into hilly terrains that are sensitive to development but also cutting through forests which elevates the risk of landslides and rockfalls due to slope instability that may then lead to fatalities (Abu Samah, 2006). According to FAO, steep terrain, vulnerable soils, heavy rainfall and earthquake activity make large parts of Asia highly susceptible to landslides (Sassa, Mikoš & Yin, 2017). Landslides disturb the ecosystem equilibrium and degrade both the soil and landscape (Giupponi et al., 2019). Luxurious hilltop developments are quite rampant in Malaysia for its exclusivity and buyers are enticed with a never-ending list of amenities and facilities in addition to promoting it as having close proximity to various highways. These highways are constructed from rugged hills and mountain terrains by cutting through slopes which are prone to erosion as the soil is washed away as a result of uncovered surface (Isa, Shamshuddin & Khairuddin, 2017). While soil erosion is a precursor to slope failure, the latter is a serious geo-hazard which is more pronounced in Malaysia for the country experiences torrential rain in addition to having a wet and humid climate. Additionally, in the tropics, the combination of high rainfall and ambient temperature causes intense chemical weathering and formation of thick soil profiles which elevates soil erosion (Dorairaj et al., 2020). According to Huat & Kazemian (2010), slope materials are weakened after rain due to a reduction in suction, leading to slope instability. Correspondingly, this chemical weathering reduces the shear strength of slope materials, thus reducing its resisting force. Further, the destructive combination of geological conditions and climate has all the necessary elements that could lead to landslides.

Slope failures or landslides, also known as mass wasting are categorized according to the type of down slope movements of rock debris and soil, in response to gravitational stress, namely flows, slides, falls, topples, subsidence and complex (Varnes, 1978). Cruden (1991) defined landslide as the movement of slope materials down a slope under the influence of gravity. The magnitude of soil erosion and consequently, slope failure is elevated with high rainfall and erosion (Crozier, 2010). Malaysia recorded 171 landslides between 2007 and March 2016, positioning itself at the tenth place with the highest frequency of landslides according to the data from the US National Aeronautics Space Administration (Nasa) (TheStar, 2018). Approximately 76% of 21,000 landslide-prone areas are in Peninsular Malaysia, while about 3,000 and 2,000 are in Sabah and Sarawak, respectively (Haliza & Jabil, 2017). Correspondingly, the total economic loss due to landslides in Malaysia was estimated to be about US $1 billion from 1973 to 2007 (National Slope Master Plan 2009–2023).

Landslides which cause destruction in unstable hills and slopes mainly ensue as a result of water, volcanic activity and seismic activity (Haliza & Jabil, 2017). Since Malaysia is located outside the Pacific Ring of Fire, landslides here often develop as a result of water movement. According to Mokhtar (2006), rainfall and storm water activities are the main aspects that lead to landslides at hillside development in Malaysia. The penetration of excess water from rainfall coupled with the existing groundwater elevate the ground water pressure and hence activate seepage flow in the soil which then reduces slope stability due to the increase in soil moisture content (Aminudin, 2009). Hence, water has indisputably appeared to be the main inducing factor of a slope failure in absence of vegetation that leads to landslide as the water that is infiltrated into the ground will seep directly into the pore space of soil, weakening soil aggregation as the soil becomes saturated thus reducing its shear strength (Mulyono et al., 2018).

**Figure 1:**
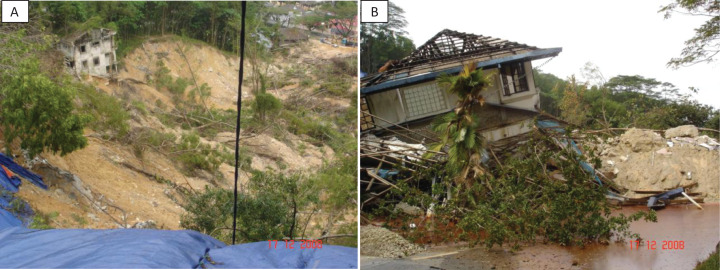
Bukit Antarabangsa landslide in 2008 which buried 14 bungalows. (A) Top view of landslide. (B) Buried bungalow. Photo credit: Normaniza Osman.

The collapse of Highland Towers, a 13-storey condominium built on a steep sloped hill in Hulu Klang, Selangor in 1993 will forever be etched in the memories of Malaysia as one of the most tragic in the country’s history of landslides. The incident killed 48 people when soil erosion at the bottom of a slope triggered the retaining wall to collapse after consecutive days of heavy downpour, while fourteen bungalow houses were completely buried in Bukit Antarabangsa in 2008 (Fig. 1) which killed five and injured fourteen (Gue & Cheah, 2008; Low, Ali & Ibrahim, 2012). These two locations are situated at the toe of the Titiwangsa mountain range and are in the same affluent neighborhood, which also boasts of being the homes of celebrities and expatriates (Ng, 2012). Fast foward to 3 years later, and yet another landslide happened in Hulu Langat, which killed 16 people, many of whom were children (TheStar, 2019). These were neither the first few nor last to have taken place at the Hulu Klang area as it is notoriously associated with constant landslides for it sits atop a hill. Heavy monsoon rain, rampant land clearing and poorly constructed retaining walls were among the causal factors of these landslides (Gue & Liong, 2007; Low, Ali & Ibrahim, 2012). Residential properties here sells like hotcake as developers entice them with scenic natural surrounding while buyers are drawn to the elite feel that is attached to each household.

Although rainfall has been one of the inevitable major causal agents of landslides, the majority of slope failures on manmade slopes occur mainly due to design failure and implementation (Jamaluddin, 2006). Similarly, 88% out of 49 landslides reportedly took place on manmade slopes (Public Works Department Malaysia (PWD), 2009). Thus, it is unsurprising that 80% of landslides occur due to human activities such as poor planning and poor slope management, agricultural activities, construction and deforestation (Chan, 1998; Shannon and Wilson, 2000). While excessive soil water content is the primary cause of slope failure, steep slopes, weak soils and topography that concentrates water contributes to landslide risk (Forbes et al., 2011).

Raj (2006) deduced that weathered granitic bedrocks in cut-slopes were weaker than consolidated materials; hence slope stability was reduced while slope morphology clearly exerted influence on slope failures (Fernandes et al., 2004). Meanwhile, Sharifah, Faisal & Shattri (2004) observed that slopes at an angle of 20–34° were more prone to slope failures in a study conducted on Cameron Highlands, Malaysia. Also, slope alteration and the placement of heavy materials on top of undercutting slopes with weak slope materials changed slope gradients and manifested a negative impact on slope failure (Chan, 1998). Understandably, steeper slopes have shallower soil profile and are exposed to rapid slope failures as they are weakly bounded. Besides, frequent slope failures were recorded on cut-slopes with heights of more than 5–10 m (Chau et al., 2002).

Nevertheless, infrastructure expansion is a necessity of any developmental projects, thus mitigation measures are necessary. The slopes earmarked for development need to be properly designed by considering the geological characteristics, structural model, local weather and soil characteristics (Song, Hong & Woo, 2012). The common approaches used in increasing slope stability are reducing slope angles, terracing and branching, improving slope drainage, the use of rock bolts and building of retaining walls whereas wire cables and wire fences are used to minimize rock falls (Wyllie, 2014). Moreover, the current practice of relying solely on engineering material or structures such as wire meshes, retaining walls, concrete and fencing are costly, less environmentally friendly, ineffective over time and inadaptable with the changing slope environment since they are not dynamic, while needing constant repair and maintenance (Aimee & Normaniza, 2014). This practice gap could arguably be addressed by applying the concept of soil bioengineering, a soft approach to slope and soil stabilization which creates minimum impact on the environment and the landscape (Bischetti, Di Fidio & Florineth, 2014). “Soil bioengineering” is a term coined to describe the application of vegetation, either parts or whole plants, specifically on low to moderate risk slopes for sustainability and stability of the slope (Coppin & Richards, 1990; Morgan & Rickson, 1992). Oftentimes, soil bioengineering and biotechnical engineering are used synonymously but the latter, sometimes also known as water and soil bioengineering (Schiechtl & Stern, 1992), involves the use of plant, or plant parts, either alone or in conjugation with inert materials such as steel, concrete and rocks for surface protection or erosion control and to enhance the soil stability (Schiechtl, 1980; Gray & Leiser, 1982). In contrast, soil bioengineering, a subset of biotechnical engineering is a multidisciplinary subject which involves the expertise of geotechnical engineering, botany, landscape architecture and hydrology (Freer, 1991; Punetha, Samanta & Sarkar, 2019). This green approach is highly sustainable as vegetation self-regenerates and could adopt and adapt to its environment, environmentally friendly, has low capital costs compared to civil engineering structures and low maintanence since the local population can be involved in the management and maintenance of the works (Giupponi et al., 2019).

There is clearly a lack of information on slope management practices in this region and the use of whole plants for slope stabilization. Thus, here we decipher into the current engineering practices including intermediate approaches for slope management. Next, is the discussion on the potential application of bioengineering, hence we cover the general attributions of a plant in slope stabilization including its mechanical, hydrological and hydraulic effects. Subsequently, we focus on species selection, and engineering properties of vegetation including rooting architecture and form and functions of root systems. Finally, the review will present the ecological principles for vegetation establishment with an emphasis on adopting the mix-culture approach as a slope failure mitigation measure. The paper should be of particular interest to readers in the areas of soil conservation and management, ecophysiology and bioengineering.

## Survey Methodology

For the compilation of this article, we did exhaustive literature search on Web of Science, Google Scholar, Science Direct, Mendeley and the University’s databases for journals, books and proceedings through the use of short phrases such as “slope stabilization methods”, “bioengineering for slope stabilization”, “causes of slope failure”,“slope management in Malaysia”, “soil bioengineering in Southeast Asia”, “use of vegetation for slope stabilization”, integrated by the usage of “+”, “vs”, “AND”, “NOT” for specific search returns. We focused mostly on reviewing the works from the past 20 years with a focus on slope stabilization practices in Malaysia and Southeast Asia in general. For references related to statistics, data were obtained from their respective websites or portals. As for literatures unavailable online and articles without open access, the service of the University’s inter-library loan and document delivery was used. Our search retrieved hundreds of publication, but only the most relevant and articles written in English were used.

### Current slope management practices and remediation methods

#### Geotextile

Geotextiles, a form of simulated vegetation is often used to temporarily or permanently stabilize soil as it mimics the properties of natural vegetation while not needing time for establishment; it provides immediate erosion control and slope stability (Álvarez-Mozos et al., 2014; Bhattacharyya et al., 2008; Saengrungruang & Boyd, 2014; Smets & Poesen, 2009). The biggest advantage of using geotextile could be the synergistic relationship it has with vegetation for it may give “composite” erosion control (Rickson, 1995). Geotextiles used for slope protection could be made of natural or synthetic material and covers blankets, nets or mats made from woven or nonwoven natural materials such as straw, jute and coir, or synthetic, for instance, polypropylene or polyester materials (Rickson, 2006). These mattings play multiple roles; hold soil in place by absorbing and holding moisture near the soil surface, promote seed germination, protect young vegetation, thwart erosion of seed, prevent wind dispersal of seed or mulch and permit for easy seed establishment (Department of Irrigation & Drainage Malaysia, 2010). Moreover, geotextiles can store runoff and trap sediment (Krenitsky et al., 1998; Mitchell et al., 2003) by lowering runoff flow velocities (Ziegler & Sutherland, 1998) and lessen the kinetic energy of raindrops and stop surface soil particles from being splashed away (Ziegler, Sutherland & Tran, 1997). Above all, the outstanding characteristic of geotextiles is its flexibility and its ability to drape as they can adhere to the soil surface after fitting, more so if the material is wet enabling it to expand (Sutherland & Ziegler, 1995).

According to Niroumand et al. (2012), geotextile is widely used as tensile reinforcement and filter to stabilize steep slopes in residual soil and weathered rock or embankments in Malaysia. It prevents soil movement or internal erosion within the slope while reinforcing the soil along potential sliding planes (Kim et al., 2019). Moreover, geotextiles could lower the pore water pressure within the slopes during rainfall, thus increasing its shear strength (Gofar & Hanafiah, 2018). Besides, the use of geotextile in cut slopes where the soil is composed of weak materials (Niroumand et al., 2012) has proven to be beneficial as the material helps to transfer the excessive shear stress from weak soil to tension in geotextile. Furthermore, the factor of safety (FOS) increases with the use of geotextile as steeper slope can be constructed to gain more space. On the other hand, Lee & Douglas (2012) explored the use of geotextile tubes on the east coast of Peninsular Malaysia, most notably in Terengganu for shoreline management. Due to the impact of high energy waves, severe erosion takes place on the mud and sandy coasts. Hence, tubes made of high strength woven geotextile are filled with sand slurry to arrest erosion that is evident with a significant increase in sand deposition on the foreshore region. Separately, Omar et al. (2019) investigated the use of natural fibers from pineapple leaves and luffa in combination with bio-grout from vegetable waste for erosion control. The former controlled surface erosion and reduced soil loss while the combined application of natural geotextile and bio-grout provided an invaluable solution for slope protection against erosion. Similarly, Chow et al. (2019) reported that water hyacinth fiber mat tested at 30° slopes under simulated rainfall with constant intensity reduced sedimentation volume by 79% compared to bare soil. However, geotextiles are costly and may not be suitable for excessively rocky sites while needing the service of an expert for installation to ensure it assists in soil stabilization and erosion control. Further, synthetic mats such as plastic sheets result in severe runoff and are easily torn and vandalized.

As most nations move towards achieving SGD goals, the use of synthetic fibers which accounts for almost 98% of all geotextiles is not justifiable though it may be robust and highly durable (Daria, Krzysztof & Jakub, 2020). These fibers are not subjected to biological degradation (Sülar & Devrim, 2019) and thus become pollutants while its production is heavily dependant on the non-renewable sources of fossil fuels (Daria, Krzysztof & Jakub, 2020). On the other hand, the use of natural fibers though seem harmless to the environment, could still end-up polluting the environment since they go through chemical treatment during the processing and production states. Although plant fibers are biodegradable (Rana et al., 2014; Sarikaya, Çallioglu & Demirel, 2019), chemical heterogeneity and their varied dimensions in plant fibers directly affects their mechanical properties (Bismarck, Mishra & Lampke, 2005). One of the inherent disadvantages of plant fibers is its tendency to degrade and decompose faster when it is exposed and is in direct contact with soil surface (Arshad et al., 2014). This is a strong indication that these fibers provide soil microorganisms the much needed source of nutrients (Daria, Krzysztof & Jakub, 2020). Currently, the use of biopolymers such as polylactide (PLA) as geotextile is gaining track for it is widely available, degradable and is competitively priced (Prambauer et al., 2019). However, the main limitations are its brittleness and stiffness, which makes it impractical to be used in slope stabilization at its current state (Daria, Krzysztof & Jakub, 2020)

### Mulching/ground cover

Mulch refers to non-vegetative material that is used to protect the soil during the critical period of vegetation establishment (Lee et al., 2018) and according to Jordán, Zavala & Muñoz-Rojas (2011), mulching is the agronomic practice of leaving mulch on the soil surface for the conservation of both soil and water which favors plant growth. Mulches are basically used temporarily to protect soil surfaces from the three main erosive agents, namely, rainfall, wind and runoff (Morgan & Rickson, 1995; Blavet et al., 2009; Jordán, Zavala & Gil, 2010). Nevertheless, at times, mulching can also be used permanently to stabilize cleared or freshly seeded areas (Huat, See & Ali, 2004). Primarily, it reduces rates of water and soil loss (Jiang et al., 2011; Liu et al., 2012; Prats et al., 2014; Prosdocimi & Cerdà, 2016; Sadeghi et al., 2015). Mulching, also a soil management strategy, increases infiltration capacity (Jordán, Zavala & Gil, 2010; Wang et al., 2016), conserves moisture by increasing water storage (Cook, Valdes & Lee, 2006; Mulumba & Lal, 2008) and reduces evaporation (Vanlauwe et al., 2015; Groen & Woods, 2008; Hayes, McLaughlin & Osmond, 2005), of soil, and aids the growth of planting materials by holding the seeds, fertilizers, and topsoil in place until growth occurs (Department of Irrigation & Drainage Malaysia, 2010). Further, mulches reduce overland flow and nutrient runoff due to increased roughness (Cerdà, 2001; Gholami, Sadeghi & Homaee, 2013), enhances organic matter content in soil through the gradual breakdown of mulches (Garcia-Orenes et al., 2009; Jordán, Zavala & Gil, 2010) while improving the topsoil temperature to promote seed germination and root development (Riddle, Gillespie & Swanton, 1996; Dahiya, Ingwersen & Streck, 2007). Mulches range from organic materials such as straw, wood chips, bark or other wood fibers and inorganic materials such as plastic sheeting, decomposed granite, rocks, and gravel (Department of Irrigation & Drainage Malaysia, 2010) and are oftentimes used in combination of mats and gluing agents.

Due to land scarcity and the expansion of oil palm cultivation, oil palm plantations have moved to steep hilly terrains. This exposed area experiences heavy losses of soil, nutrients and organic matter (Ghulam, Yusoff & Cyril, 1997). Ping et al. (2012) successfully utilized empty fruit bunches (EFB) and Ecomat to reduce soil erosion on sloping lands. Ecomat is a biodegradable mat made of oil palm fibers used as mulch on hilly slopes (Khalid & Tarmizi, 2008). The use of EFB as a mulching material is commonly practised in oil palm estates in Malaysia. Both EFB and Ecomat improved soil organic matter, soil nutrient contents and humic substances by improving soil aggregate stability and aggregation (Khalid & Tarmizi, 1999; Khalid, Zin & Anderson, 2000; Khalid & Tarmizi, 2008; Ping et al., 2012). Moreover, pruned oil palm fronds, used as mulching agent, are often stacked along palm avenues across the slope. This practice had managed to reduce soil run-off by 13% and soil erosion to less than 5 t/ha^−1^ per year (Soon & Hoong, 2002). Nevertheless, there are limitations to the use of mulch as a soil stabilizer. It cannot be used as a permanent soil cover and needs to be removed after plant establishment. Mulches employed on steep slopes should be secured with netting while thick mulches may lower soil temperature, hence delaying seed germination (Qu et al., 2019). In addition, the use of certain mulches such as wood chips could absorb nutrients that are essential for the growth and development of plants (Griffin, Reid & Bremer, 2007; Maggard et al., 2012). Nonetheless, mulches are prone to erosion and may be washed away during a storm or heavy downpour thus needing periodic maintenance to ensure that mulches present an effective erosion control.

### Live poles

Live stakes or live poles are stem cuts from trees or shrubs which are installed vertically or in a direction perpendicular to the slope (Wu, 2007). It is often used for shallow slope stabilization or in other words, combat shallow slope failure within 1–2 m (Wu et al., 2014; Boldrin, Leung & Bengough, 2017; Liang et al., 2017). Pole transpiration-induced suction can lower soil hydraulic conductivity and rainfall infiltration (Ng & Leung, 2012; Ng, Leung & Woon, 2013; Ng et al., 2016; Leung et al., 2018, Leung, Garg & Ng, 2015) which increases the shear strength of the soil. These live poles provide reinforcement of slope shoulders, serve as horizontal drainage, act as surface flow retardation and barriers to earth movement to control slope erosion (Mafian et al., 2009; Prasad et al., 2012). It has been reported that the use of live poles of *Dillenia suffruticosa* and *Hibiscus tiliaceus* significantly increased the factor of safety on slopes of residual tropical soil with the inclination of 28–29° as a result of improved mechanical strength (Mafian et al., 2009; Prasad et al., 2012). The general drawback of this method is soil disturbance during installation.

### Brush layering

The technique of laying live cuttings or pieces of brush on horizontal benches that follow the contour of either an existing or filled slope is known as brush layering (Eubanks & Meadows, 2002; Bischetti et al., 2010). Ultimately, it is a layer of plant intercepted between layers of soil on cut or fill slopes. These layers often serve as earth-reinforcing units to provide shallow stability of slopes (MacNeil et al., 2001) and act as live fences to capture debris and continuous shallow raking drains (Barker, 2001). In addition, brush layering also improves the infiltration and drainage of wet slopes (Lewis, 2000) while the stems of the cuttings extend into the hillslope and act as tensile reinforcements (Bischetti et al., 2010). Contrary to the design parameters reported by Gray & Sotir (1996) and Morgan & Rickson (1995), the modern approach by Bischetti et al. (2010) introduced a new design for brush layering based on equilibrium limit equations and by considering brush layer design parameters, namely, number of stems per meter, length and diameter of stems and distance between brush layers. Based on the calculation of FOS, Bischetti et al. (2010) reported that by using half of the live materials typically involved in this technique, the same stabilization can be obtained with a great saving of cost and time.

However, brush layering is only apt for use when slope failure is predicted to take place while the live plant has to be given adequate time to acquire sufficient strength to fully stabilize soils.

The delay to acquire adequate strength by vegetation is an inherent limitation of soft engineering structures. Although the construction of brush layering is simple and fast, it requires more excavation compared to live staking and live fascine methods (Donat, 1995) and is deemed unsuitable for rocky slopes. Moreover, brush layering can be comparatively expensive and labor-intensive especially when large amounts of backfill are needed (Alaska Department of Fish & Game, 2005).

### Rock buttress

Rock buttress or rock fill is a fill rehabilitation method on an unstable slope to reduce erosion from rainfall (Ahmad, Mohammad Zaki & Ayob, 2016) especially if adequate rock fills are available locally. This method is based on a simple approach to increase slope stability by increasing the weight of the material at the toe by placing weighted large stone materials, which increase the resisting forces while resisting failure (Chatwin et al., 1994; Shannon and Wilson, 2012). The practice of placing the rock against the slope face adds to stabilizing force while reducing the overall slope height (Saftner, 2017). Though this is a common mitigation measure used in Malaysia due to its low cost, there are no publications or official reports made available to the public. Nevertheless, Ghazali, Mdyusoff & Azmi (2019) reported slope failure as the result of rock fill along Temerloh-Maran Expressway in Peninsular Malaysia. The main disadvantage of this method is the rock fill adds weight to the slope hence increasing slope stress which then leads to slope failure due to slope instability.

### Concrete structures

Though very costly, concrete structures remain the popular choice in Malaysia due to durability and the availability of high-quality raw materials. Among them, retaining walls, namely crib wall, gabion, rubble and earth wall are used as slope stabilization structures to fix excavated slopes and road embankments. The principle of this method is to apply a retaining structure to withstand the downward forces of the soil mass (Mizal-Azzmi, Mohd-Noor & Jamaludin, 2011). Although sturdy, these structures had failed on numerous occasions as the materials are highly susceptible to degradation, especially when quality assurance measures are not monitored during the construction stage. For instance, Penang Island has experienced countless slope failures which included collapsed concrete structures installed along Tanjung Bungah, a hillslope area (Yahaya et al., 2019). The fill material used was deemed unsuitable for it was made up of sandy clay and clayey silt that was highly permeable which led to saturation and increased pore water pressure in the embankment which resulted in the failure of its retaining wall (Department of Mineral and Geoscience Malaysia (JMG), 2017). In general, concrete structures lack esthetic value while the white grayish concrete proved to be an eyesore as the public becomes more environmentally conscious. The public prefers to look at greeneries as opposed to inert structures. Moreover, these structures prohibit the growth of plants on slopes and therefore give very low ecological values (Leung et al., 2015).

### Use of vegetation: the way forward

#### Role of plant—a tribute

**Figure 2:**
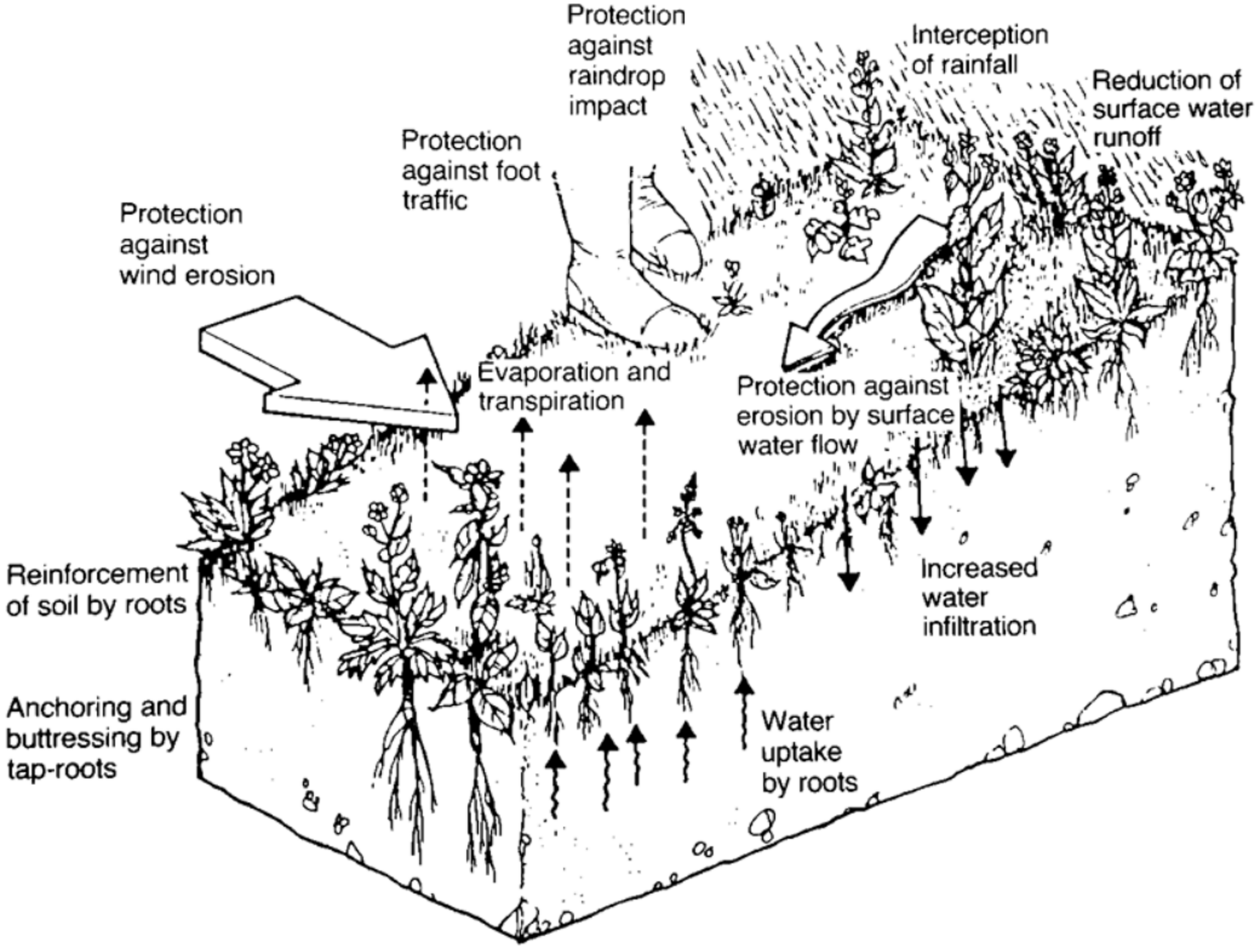
Role of vegetation (Source: Coppin & Richards (1990), © CIRIA).

The green approach of using plants for the alleviation of slope instability has been practiced worldwide. Likewise, the contribution of the two plant aspects namely hydrological and mechanical aspects are widely discussed from both aboveground and belowground attributions (Fig. 2). Vegetation cover increases the soil shear strength employing its root network through mechanical reinforcement, anchoring and compaction (Singh, 2010). Moreover, cover crops guard the soil surface against the impact of rainfall by decreasing the erosive capacity of the flowing water by lowering its velocity (Rey, 2003) whilst restoring slope physical condition. Meanwhile, plant litter shields the soil surface from raindrop impact and slows the movement of water across the soil surface. Besides that, plants play a crucial role in reducing the moisture content of soil through evapotranspiration which allows the soil to absorb more water. Also, the utilization of vegetation to enhance slope stability is governed by the type of plants used, the planting technique and root properties (Huat & Kazemian, 2010). It has become an alternative approach for slope stabilization against erosion besides minimizing the incidence of landslides (Normaniza & Barakbah, 2011; Liu et al., 2016).

In Malaysia, re-vegetation of cut slopes along the highways involved plant selection followed by research on the gully erosion control and vegetation establishment on degraded slopes (Noraini et al., 2000). However, the technique relied on cut stems for its coppicing abilities and the soil binding properties of roots into civil design (Noraini & Jasney, 2001). In this section, we will discuss the potential use of whole live vegetation as a soil and slope stabilizing structure. Vegetation can be regarded as “soft” engineering structure as it protects the soil surface from erosion through mechanical, hydrological and hydraulic effects.

### Mechanical effects

Roots with its finger-like projections provide root reinforcement and strong anchorage that binds the soil particles together to prevent the collapse of soil structure. On slopes, vertical roots that elevates the pullout resistance (Anisuzzaman, Nakano & Masuzawa, 2002) may break through the entire soil mass, anchoring into more stable layers and increasing resistance to sliding whereas dense lateral roots stabilize soil surface layers against landslides (Sidle et al., 2006). In other words, roots growing perpendicular to the soil surface provide resistance to shearing forces acting on the soil whereas those extending parallel to the soil reinforces the tensile strength of the soil zone (Jerome, 2010). Generally, roots provide mechanical strength to the soil through its tensile strength, adhesive and frictional properties (Reubens et al., 2007). Root properties such as the number of roots, tensile strength, size and bending stiffness determine slope stability (Reubens et al., 2007). Meanwhile, the degree of soil reinforcement is not only regulated by tensile strength and root density but plant cell wall components such as lignin, cellulose and hemicelluloses, the length to diameter ratio, orientation and bending stiffness of roots penetrating the failure planes (Reubens et al., 2007; Saifuddin et al., 2015). According to Normaniza & Barakbah (2006), the highest root length density (RLD) was detected in a stable slope with the highest density of vegetation which resulted in lower water content (SWC). Besides, RLD was positively correlated to shear strength while SWC was inversely related to both soil penetrability and shear strength.

### Hydrological effects

The hydrological effects of vegetation cover are evident through the reduction in water runoff by establishing the water cycle of soil-plant-atmosphere continuum (SPAC) and ensuring the slope is relatively dry (Normaniza & Barakbah, 2006; Mafian et al., 2009; Normaniza, Saifuddin & Halim, 2014). It is more pronounced with the reduction of soil water content by means of transpiration and interception of precipitation (Greenway, 1987). As the roots function by regulating the soil water content from exceeding its field capacity (Normaniza & Barakbah, 2006) while absorbing and circulating the water to the atmosphere rather than letting all infiltrates deep into the soil (Abdullah, Nomaniza & Ali, 2011), the plant canopy lowers the effective precipitation and erosion effect on a slope’s surface by intercepting rainfall (Zhao et al., 2019). According to Seitz & Escobedo (2011), rainfall interception varies with plant type, plant canopy and planting density. In addition, the aboveground biomass acts as a buffer that reduces the velocity of raindrops hence reducing its kinetic energy and preventing splash erosion by reducing big raindrops into smaller raindrops (Marc & Richard, 2009). The depletion of soil moisture as a result of root absorption induce the soils to crack (Mulyono et al., 2018) thus the rate of infiltration is increased in presence of vegetation which then reduces run-off as more water is removed by evapotranspiration from the soil (Noraini & Roslan, 2008). Infiltration is the process of water movement from the ground surface to the soil via gravitational force (Ghestem & Sidle, 2011). Further, Dohnal et al. (2009) observed that macropores created by the penetration of roots which enhanced the soil porosity played a major role in increasing the infiltration rate.

### Hydraulic effects

The striking hydraulic effect of vegetation is the reduction in flow capacity due to the contact between the plant and flowing water (Noraini & Roslan, 2008). On the other hand, the attribute of roughness is contributed by the stem and roots that limit the capacity of flowing water, hence limiting the detachment and transportation of soil sediment (Mulyono et al., 2018). Besides, the presence of vegetation leads to a reduction in the inertial force of the surface runoff while the water flow around the vegetation increases the viscous force (Zhao et al., 2019). Further, vegetation restricts the surface runoff from spreading along an entire slope’s surface. In addition, the hydraulic mechanism of vegetation is manifested through pore-water pressure reduction in soil by root water uptake (Ng, Leung & Ni, 2019), resulting in a reduction in permeability, but an increase in the soil shear strength (Liu et al., 2016).

### Types of vegetation

#### Grasses

Grasses offer short-term protection against surface erosion and minor protection against shallow slope failures. They are quick growing and possess a dense network of shallow roots that offer protection against surficial erosion (Gray & Sotir, 1996). However, grasses are short-lived and its use requires regular maintenance while hand planting is labor intensive and expensive (Coppin & Richards, 1990). Moreover, they lack the ability to grow during dry season whereas the seeds get washed off in the event of heavy rainfall. Nevertheless, *Vetive*r sp. exhibits deep root systems and is often used in the restoration of eroded or unstable slopes (Stokes et al., 2008). *Chrysopogon zizaniodes*, is a widely planted *Vetiver* sp. for soil and water preservation, land rehabilitation, and embankment stabilization (Rahardjo et al., 2014). Its deep rooting allows the plant to fetch water from the soil and stabilize the slopes. The ability of this grass to adapt and grow in different climatic conditions makes it highly valuable for reinforcement work (Rahardjo et al., 2014).

#### Herbaceous

Herbaceous plants usually possess more diffuse or fibrous root systems than those of woody plants (Stokes et al., 2009). The fibrous roots possess more fine and thin roots compared to woody species, hence the root area ratio is higher while the tensile root strength is comparable to roots from woody species (Mattia, Bischetti & Gentile, 2005; De Baets et al., 2008; Loades et al., 2013). They grow closer to the ground, providing dense ground coverage with a shallow root system (Stokes et al., 2008). Herbaceous legumes are nitrogen-fixing plants that grow well in presence of grasses but planting material such as seeds may be expensive while seedling establishment is difficult on harsh conditions (Coppin & Richards, 1990). In Malaysia, *Arachis pintoi, Wedelia trilobata* and *Pandanus pygmaeus* are commonly planted as ground cover.

#### Woody plants and shrubs

Woody plants provide greater protection against shallow slope failures compared to herbaceous vegetation. These types of vegetation modify the soil moisture regime via evapotranspiration and grant root reinforcement within the soil mantle (Stokes, 2000). Shrubs are low-growing woody plants with multi-stems that may be as short as 0.2 m or grow up to 6 m in height. They don’t grow as tall as a tree, thus it is easier to control and maintain (Stokes et al., 2008). Though the roots cannot penetrate as deep as a tree, its tensile strength is comparable. According to Tosi (2007), the roots of pioneer shrubs namely *Rosa canina, Inula viscosa and Spartium junceum* possess comparable tensile strengths to tree species such as *Quercus, Pinus, Picea* and *Salix* which echoed the findings of Leung et al. (2015) which reported the root reinforcement effects of shrubs were comparable to trees. However, these shrubs species do not exhibit the negative effects often attributed to the dynamic and static surcharges of large trees but are able to increase the soil shear strength due to the presence of thin roots that exert maximum tensile strength during soil displacement. Orange Jasmine (kemuning), *Murraya exotica* L., a native of South East Asia, is a tropical evergreen shrub that flowers throughout the year (Rahardjo et al., 2014). According to Francis (2003), seedlings quickly develop deep root systems while Rahardjo et al. (2014) reported that it minimized the infiltration of rainwater into slopes, increased soil shear strength and maintained the negative pore-water pressure during rainfall. These makes it an ideal potential slope plant in addition to the following list of suitable slope plant species recommended by the Department of Irrigation and Drainage Malaysia (2010): *Cassia biflora, Caesalphina pulcherrima, Dillenia suffruticosa, Dillenia indica, Hymenocallis littoralis, Heliconia* spp., *Mussaenda eryhrophylla “Dona luz”, Melastoma malabathricum*.

#### Trees

Trees are mostly evergreen and perennial having a main stem with the roots growing several meters deep and wide (Stone & Kalisz, 1991). Though trees are suitable for soil buttressing on slopes, tall and large trees are highly vulnerable to falling during storms especially if the soil is shallow, hence reducing slope stability (Stokes et al., 2008). Trees reinforce the soil matrix through their root system, by improving soil shear strength (Operstein & Frydman, 2000), providing structural support and lowering the pore water pressures in the soil (Coppin & Richards, 1990; Gray & Sotir, 1996; Genet et al., 2008). The Department of Irrigation and Drainage Malaysia (2010) has listed the following as suitable erosion control tree species: *Andira surinamensis, Cassia surattensis, Cassia fistula Rajah, Cassia spectabilis, Fagraea fragrans, Khaya senegalensis, Millettia atropurpurea, Peltophorum pterocarpum*.

### Selection of plant species

The selection of live planting material is vital as it should meet certain criteria, such as the ecological make-up of the species, biotechnical aspect, its origin, age and plant size (Schiechtl & Stern, 1992). The main limiting factor of the application of soil bioengineering is climate, since it influences the physiological development of roots (Zhong, Liang & Ting, 2009) that reinforces the soil through mechanical and hydrological mechanism while slope plant establishment varies between different geographical areas (Alday, Marrs & Ruiz, 2010; Burylo, Rey & Delcros, 2007; Florineth, Rauch & Staffler, 2002). Hence, the plant species selected must be adapted to its environment in terms of abiotic factors such as water, nutrient, light and temperature as it is essential to guarantee the success of bioengineering practices for slope stabilization (Stokes et al., 2014). Among others, stem density, stem bending resistance, root density, root area ratio, the potential to trap sediment and debris, root tensile strength and root morphology are traits of importance (Baets et al., 2008; Stokes et al., 2009; Giadrossich et al., 2012; Bischetti, Di Fidio & Florineth, 2014; Ghestem et al., 2014). The list could be extended to high photosynthetic rate, transpiration rate, growth rate and rooting parameters such as high root biomass and high wood components, namely, cellulose and lignin (Normaniza, Faizal & Barakbah, 2008; Saifuddin & Normaniza, 2012). The following criteria are based on available literatures:

- The presence of both extensive deep-rooted (e.g., *Leucaena leucocephala*) and shallow-rooted (e.g., *M. malabathricum*) profiles of slope plants or grasses are preferred as different root architectures also contribute to different protection and stabilizing function (Yen, 1987; Coppin & Richards, 1990; Greenwood, Norris & Wint, 2004).
- The plant should be fast-growing and self-sustainable since a fresh cut slope is bare, infertile and eroded. Leguminous plants (e.g., *Pueraria javanica* and *Calopogonium mucunoides*) are fast growing and also could self-sustain on the barren soil due to high capacity in nitrogen fixation ability.
- To encounter the ever-rising carbon dioxide level in the atmosphere, the structural and functional aspects of the plants viz. large canopy, large leaf area and density of plant cover, for example (*D. suffruticosa*) could be accounted for providing an avenue for carbon sequestration. Thus, the carbon sink potential of slope plants is essential for the environmental and slope sustainability aspect (Normaniza, Saifuddin & Halim, 2014; Normaniza & Barakbah, 2008).
- The slope plant should thrive and be resilient in a broad range of climatic and soil conditions
- Drought tolerant plants are much sought after as in Malaysia, in addition to intense rainfall, the country experiences “transient drought” or irregular month-long dry periods. *Lantana camara* for instance, can withstand drought by exhibiting smaller leaf areas, suppressed growth and longer root length.
- The use of flowering plants is recommended for the colorful flowers could attract the fauna (e.g., bees, butterflies, insects) to come into the plant community and flourish the slope ecosystem. For example, the combination of *M. malabathricum* (purple-pink), *Hibiscus rosa-sinensis* (multi-coloured) and *L. camara* will provide a scenic view along the highways for they are not only beautiful but resilient and provide value-added esthetic values via ecological and safety attributes to the environment and mankind.

In addition, the following are points for consideration:

- The rooting architecture may change overtime, for instance, oaks and conifers possess tap and sinker roots when young, however as they mature, these plants develop shallow root system and thus signaling the end of rein of the tap and sinker roots.
- The plant canopy could play a big role in rainfall interception. Although evergreen plants with dense leaves look like a clear winner, deciduous plants should not be overlooked. Some may give equal protection to that of evergreens.
- There should be a compromise between the growth of plant canopy and roots. Slope areas prone to deep-seated failure may be planted with shrubs instead of trees due to its limited exposure to wind and weight. (Gray & Leiser, 1989).
- The choice of plant should aim at the establishment with minimal maintenance.
- Always opt for plants that grow in similar habitat.

### Native plant species

In principle, indigenous or native plant species are preferred in place of introduced or alien species (Ghestem et al., 2014). These plants are better acclimatized to the local condition and environment, thus they are often deemed sturdy and competitive (Gray & Sotir, 1996). Moreover, they might have the ability to co-exist with its pathogen or less susceptible to disease. Besides, once established very little care goes into maintenance such as irrigation and fertilization while blending esthetically with the ecosystem. According to Stokes et al. (2009), usage of native plants could increase the success rate of planting while reducing long-term maintenance. However, the availability of planting material such as seeds and seedlings could be limited due to the lack of propagation methods. Conversely, native plants come with a narrow range of plant species for selection, more so for eroded slope areas.

### Introduced or exotic plant species

Introduced or exotic plant vegetation comes handy due to its bigger planting reservoir and commercial availability. In some cases, these introduced species may be better suited to the local area due to random chance in evolution or evolutionary changes (Gray & Leiser, 1982). For example, in Malaysia, introduced tropical plants, *L. leucocephala* and *Peltophorum pterocorpum* are grown on slopes since the extensive root growth provides high root tensile strength and soil shear strength which provide long term soil reinforcement on slopes (Normaniza, Saifuddin & Halim, 2014).

### From introduced pioneers to established slope ecosystem

Both grasses and legume creepers or trees are potentially good slope pioneers as they need to fix the quality of the soil to initiate the succession process. Although grass shows 20–50 times lower nitrogen-fixing capacity than those by legume species, the nitrogen enhancement capacity of both grasses and legumes are evident when they are grown together as slope pioneers. Equally important is the choice of suitable pioneer species that can fasten the process of natural succession (Bardgett & Walker, 2004) since poor selection could disrupt the entire process. Likewise, the existence of an initial plant cover is imperative in initiating the process of stabilization and build-up of organic material (Bradshaw, 2000; Parrotta & Knowles, 2001; Nicolau, 2002).

Natural plant succession starts from initial pioneer vegetation; therefore the pioneer species should possess good characteristics such as fast-growing capacity, nitrogen-fixing, self-sustainability, good plant water relations and extensive root growth (Normaniza, Faizal & Barakbah, 2009). Woody species plays a key role in succession by serving as a bridge between herbaceous colonizers such as grasses and legumes for the restoration of problematic sites (Polster, 2003). Leguminous plants are a natural choice for its ability to fix nitrogen and rehabilitate infertile soils. The evergreen *L. leucocephala*, a leguminous tree that is found abundantly throughout the tropics including Malaysia is known to be versatile pioneer species that is used as a potential slope plant due to its erosion control ability (Parera, 1983; Duke & DuCellier, 1993; Normaniza & Barakbah, 2011). According to Normaniza, Faizal & Barakbah (2009), based on 2 years of observation, this species accelerated the plant succession and the revegetation process when grown on newly cut slopes. The plant permitted the influx of new plant species amounting to 46 in a mix-culture approach in addition to monoculture while sustaining amid the competition for water, light, nutrients and space.

**Figure 3:**
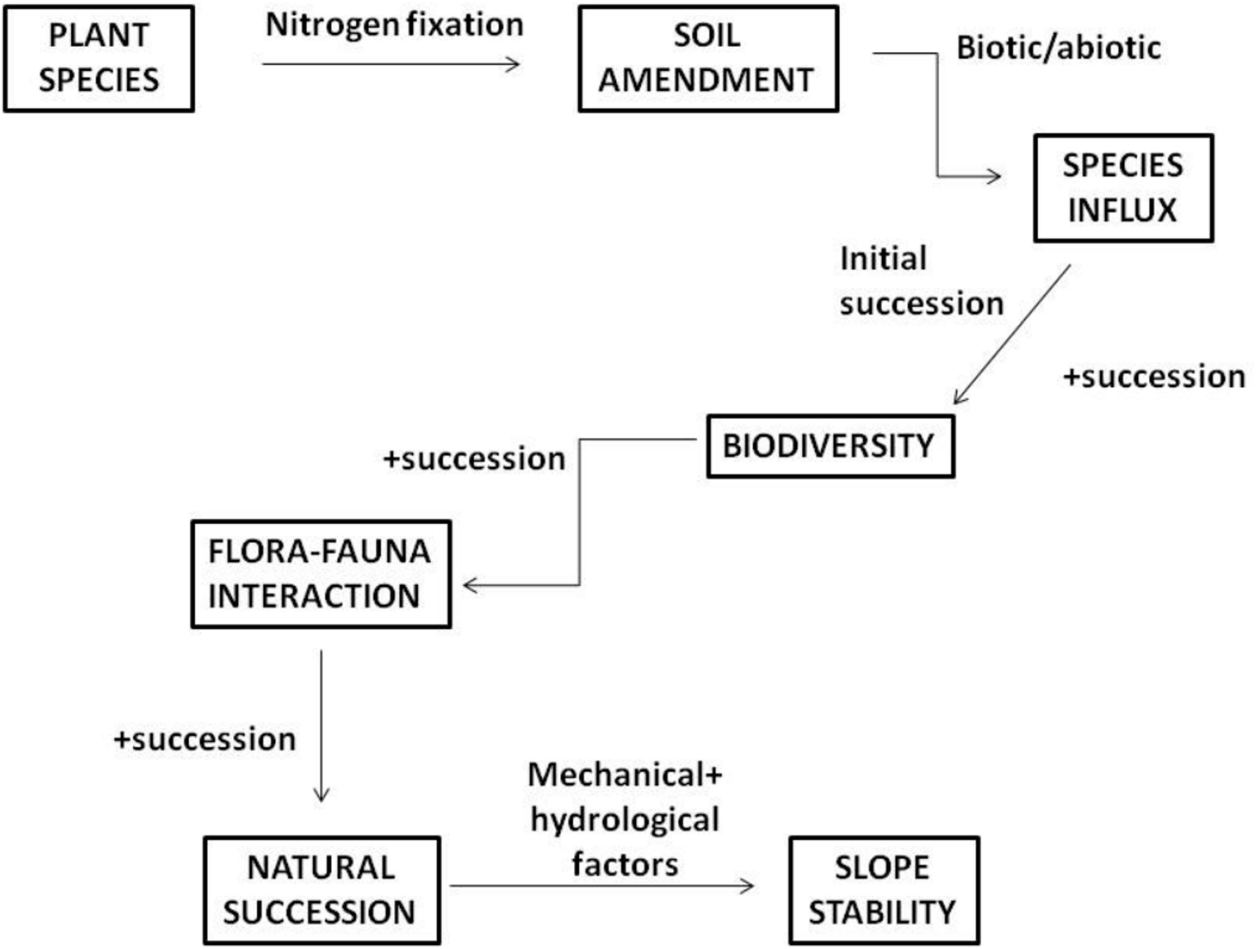
Slope stability mechanism to hasten natural succession (Normaniza, Saifuddin & Halim, 2014).

Normaniza, Saifuddin & Halim (2014) proposed a mechanism to enhance the process of natural succession for slope stabilization by placing the priority on the selection of the right pioneer species (Fig. 3). Ideally, it should be a nitrogen-fixer since barren slopes are infertile and unsuitable for the healthy growth of plants in general. Due to high rainfall, the soils of the tropics, are highly weathered, leached, acidic and low in base saturation (Foy, 1992), contains high levels of organic matter and very low mineral contents (Snyder, Jones & Gascho, 1986). In such state, soil amendment is the way to go as it offers a quick solution to increase the soil pH and provide the plant with the much needed nutrients. This soil remediation method would then allow the initial succession process to take place through the changes of abiotic and biotic factors. Subsequently, the influx of new plant species will not only enrich the plant biodiversity of slopes and accelerate the process of natural succession, but attract pollinators to the new ecosystem. This flora-fauna association promotes seed dispersal, which would ultimately enhance the natural plant succession process. Ultimately, the mechanical and hydrological aspects of vegetation aid in the attainment of slope stability.

### Form and functions of root system for slope stability

Root-soil matrix is an integral component of soil stabilization as roots are strong in tension while soils are strong in compression and this “yin-yang” like complementary interaction results in a reinforced soil (Sanchez-Castillo et al., 2017). During soil shearing, the roots project their tensile strength whereas shear stresses that develop in the soil matrix are transferred to the root fibers via tensile resistance of the roots (De Baets et al., 2008). Roots enhance soil shear strength and residual strength through reinforcement of soil structure. While the former is highly dependent on root distribution, branching pattern and root density (Saifuddin et al., 2015), the roots could increase the reinforcement by growing across failure planes into deeper stable soil layers and acting as piles (Mattia, Bischetti & Gentile, 2005; Morgan, 2007). In a pull-out strength test, the tensile strength was negatively correlated to root diameter (Ali, 2010). It was reported that amongst the species tested, the highest root tensile strength was observed in *L. leucocephala*, followed by *A. mangium* and *M. malabathricum*. The observation is postulated to be the result of the presence of long tap and extensive lateral roots in *L. leucocephala*. Meanwhile, the ability of roots to take up water from the soil is strongly influenced by the amount of water within the soil, matric potential of the soil, length of roots in the soil, the specific activity of the roots and the placement of roots within the soil (MacNeil et al., 2001).

In bio-engineering, shear strength is exhibited in the form of shearing resistance by the roots as they physically bind or restrain soil particles, resulting in friction and interlocking between the root and soil particles while elevating the level of soil cohesion (Mickovski & van Beek, 2009). Abdullah, Nomaniza & Ali (2011) reported among the three potential slope plants tested, *Acacia mangium* had the highest shear strength values, 30.4 kPa and 50.2 kPa at loads 13.3 kPa and 24.3 kPa, respectively while *L. leucocephala* exhibited the highest cohesion factor, which was almost double the value of *D. suffruticosa* and *A. mangium*.

For the enhancement of slope stability, it should ideally contain both fine and coarse roots, the latter can be broken down into four classes, namely, taproot, lateral roots, basal roots and adventitious roots (Schwarz et al., 2009). Fine roots (1–2 mm) are highly efficient in stabilizing the top soil layers for they possess higher tensile strengths while coarse roots (2 mm) aid in anchoring large volumes of soil as they extend into greater depths of the soil (Jerome, 2010). Besides, the coarse roots are more rigid and possess a higher bending stiffness to withstand greater bending stresses than fine roots. Since tensile strength is inversely proportional to root diameter, if a plant possesses a higher number of fine roots, it will provide better soil reinforcement. Moreover, though fine roots tend to break off, it will remain within the soil in the event of a slope failure unlike coarse roots which can slip out (Jerome, 2010). In addition to fine and coarse roots, Stokes et al. (2009) included thick roots (more than 10 mm) into the list of root classes. These roots serve as anchors and prevent the uprooting of plants while the spacing of these roots determines the position of the fine and thin roots in the soil, and hence indirectly influence nutrient and water uptake. Generally, the depth and root architecture are highly responsive and influenced by environmental conditions, namely, local climate, soil fertility and moisture content (Sauter, 2013), thus displaying root plasticity.

Yen (1987) proposed a root system based on the tap, lateral and horizontal roots, classifying them into five types, namely, H, M, R, V and VH (Fig. S1). The H- and VH-root types were deemed suitable for soil reinforcement, slope protection and wind resistance. On the other hand, the M-type was effective in controlling soil erosion while the V-type was suitable for wind resistant (Reubens et al., 2007). R-type root architecture is favorable in protecting slope from failure and was found to be more effective than V-type root in improving soil shear strength (Fan & Chen, 2010). Based on a study by Saifuddin & Normaniza (2016) (Figs. S2 and S3), the root systems of *A. mangium*, *L. leucocephala* and *D. suffruticosa* are VH-, H- and M-types, respectively. Thus, *A. mangium* and *L. leucocephala* are suggested to be planted in the middle of a slope as the deep penetration of tap root could intersect the shear plane and reduce the shear plane movement while *D. suffruticosa* which possess shallow roots is planted at the toe or top of the slope where roots increase the cohesion at the end of shear plane (Abdullah, Nomaniza & Ali, 2011). In a nutshell, from the perspective of slope stability, it is highly recommended that bigger trees are planted all over the lower third of the slope. According to Danjon et al. (2008), species with vertical and strong roots stabilizes the soil in the middle of the slope, whereas those with denser and stronger roots upslope or downslope will better reinforce the top or toe of the slope, respectively (Ghestem et al., 2014).

On the other hand, Köstler, Bruckner & Bibelriether (1968) categorized the tree roots into heart, plate/sinker and tap root systems (Stokes & Mattheck, 1996). Under the heart system exhibited by most angiosperms, horizontal and vertical laterals grow from the base of the tree. This root system provides the most efficient anchorage (Stokes et al., 2000) as it integrates and combines the rigidity provided by the trunk and dense fibrous networks further away, which subsequently improves the soil shear resistance (Wu, Bettadapura & Beal, 1988). As for the plate system, it consists of horizontal lateral roots stretching out from the base of the gymnosperms. Meanwhile, vertical sinker roots develop and grow downwards from the main lateral roots whilst trees with tap root systems have a large tap root anchoring the tree directly, like a stake in the ground with smaller horizontal lateral roots (Ennos, 1993). The tap root system is a coherent structure on sand due to the increased rooting depth (Stokes, Lucas & Jouneau, 2007). On the contrary, the heart and tap root systems are the most resistant to uprooting while plate systems are the least resistant (Norris et al., 2008). For slope stabilization, trees with deeper tap and plate rooted systems can be planted in the middle and top of a slope, respectively (Danjon et al., 2008).

### Plant diversity

#### Mono-culture

The reliance on planting a single species namely, mono-culture, is not advisable as it has exhibited a deteriorating effect on slope stabilization and sustainable slope protection (Normaniza & Barakbah, 2011; Stokes et al., 2014). The general practice is the use of hydro-seeded grasses, a short-term solution as the ground coverage is reduced over time due to the shallow root system (Normaniza & Barakbah, 2011). The top soil is then exposed to rainfall and chemical weathering that leaches off the nutrients from the soil, deeming it infertile and unsuitable for other plant species to grow and arresting the succession process. Besides, monospecific planting risks the widespread devastation in the event of disease due to the lack of tolerance and adaptability to the change in environmental conditions (Stokes et al., 2014). The worst-case scenario in monoculture is the use of alien species which may turn invasive, impeding colonization of native plants by forming dense thickets and capturing and absorbing available nutrients and resources (Walker et al., 2010). However, if left with no alternative solution, mono-culture could be practiced by increasing the plant density. Halim & Normaniza (2014) reported that the plant density was inversely related to the soil saturation level and erosion rate on the slope with an angle of 45°.

#### Mix culture

As a long-term restoration strategy and slope protection, a mix-culture system should be adopted because each plant species comes with a different rooting system which helps keep soil erosion at bay (Marden, Rowe & Rowan, 2007). Correspondingly, under 2 years of observation on a cut slope, mix-culture plots which comprised of *L. leucocephala*, *Ischaemum muticum* (grass), *Pueraria phasoiloides* (creeper) and four other slope plant species displayed fast growth rate, enhanced physiological traits and an increased plant diversity with a record of 39 new colonizers by the end of the experiment. Among others, dominant successors observed were *M. malabathricum* which covered up to 15.0% of ground cover, *Stachytarpheta indica* (shrub) and *Dieranopteris lineanis* (fern) (Normaniza, Faizal & Barakbah, 2009; Normaniza & Barakbah, 2011). It was reported that the soil penetrability and soil shear strength increased significantly in the mix-culture plots, especially in presence of *L. leucocephala* as compared to monocultures while the soil saturation level exhibited the lowest percentage amongst the four plots (Normaniza & Barakbah, 2011). Thus, it is apparent that the right pioneer plant could markedly increase the plant diversity which in turn will reduce the risk of slope failure by enhancing slope stability (Pohl et al., 2009; Genet et al., 2010). Moreover, most studies on plant diversity reported a negative relationship between vegetation coverage and soil erosion (Marques et al., 2007; Zhou et al., 2008) which ranges from a linear (Greene, Kinnell & Wood, 1994) to an exponential (Marston, 1952) correlation.

Slope revegetation is essential for restoring the physical, landscape, and ecological functions of a barren site (Kil et al., 2015). Thus, ideally, the focus ought to be on selecting the right plant mixture to be planted at the right density which will eventually create a sustainable and stable slope. Giupponi et al. (2019) suggested investing on good mixtures of pioneer plant seeds that are capable of establishment on infertile land, which is the likely scenario of slope soils. Right seed mixtures can fasten vegetation dynamics, accelerate vegetation succession and maximize the success of soil stabilization. Furthermore, the right composition of species provides a positive effect on slope soil organic carbon storage as high plant diversity tremendously enhances the soil carbon sequestration (Chen et al., 2018). It was reported that high diversity of plant species performs a high level of specialization between species, such as species-specific rooting structures (Loreau et al., 2001), implying that the pervasive impact of biodiversity on environmental processes also relates to the ecosystem service of erosion protection. Species diversity in an ecological community is beneficial to the ecosystem stability, sustainability, and rehabilitation (Xu et al., 2019) while the application of mix-cultures can mitigate climate change of terrestrial ecosystems in the short-term while encouraging a low-carbon economy in the long-term (Mackey et al., 2013), hence supporting the global Sustainable Development Goals (SGD) no. 13 and 15. In short, proper implementation of mix-culture not only hastens plant succession process, but also sustains green landscape and provides long-term slope stabilization.

### Future perspective

The “tree grasses”, bamboos, have in recent years gained renewed interest as a material for slope stabilization (Xu et al., 2019). The abundance and global distribution of this group of plants with high vegetative propagation ability in addition to the sturdy nature of its dense culms and extensive fibrous root systems, makes it ideal for slope strengthening and reinforcement works (Tardio et al., 2018; Rao et al., 2018). Besides having high mechanical and tensile strengths, bamboos are flexible and lightweight (Bhonde et al., 2014; Javadian et al., 2019). Moreover, the presence of bamboo forests in mountainous areas with very steep slopes is prove of its soil strengthening capability (Tardio et al., 2018). However due to its strong colonization ability, bamboos have high levels of invasion potential (Roy et al., 2016; Srivastava, Griess & Padalia, 2018) hence limiting its utilization in bioengineering. In addition, bamboo has low durability due to its high sugar and starch content in its culm which makes it highly susceptible to decay hence, changing its biotechnical characteristics (Tardio et al., 2018; Kaminski et al., 2016). Nevertheless, its sustainability and versatility make it a suitable material for structural applications and to be incorporated in mixed soil bioengineering work (Javadian et al., 2019).

Ideally, after the soil bioengineering work has begun, environmental monitoring should follow suit but often times, this pivotal component that is used to evaluate the effectiveness of soil bioengineering on the ecosystem and the landscape is left out due to lack of funding and poor planning (Giupponi et al., 2019). Recently, Giupponi et al. (2019) suggested the tracking and observation of vegetation and soil of the area under such work as vegetation can be a “super indicator” of environmental quality as well as an expression of the characteristics of the ground on which it lies (Cassinari et al., 2015). The analysis of the floristic-vegetational and ecological features of the plant communities and physio-chemical characteristics of the soils under the soil bioengineering intervention are highly likely to unravel key insights into the suitability of the method and provide room for improvement of soil bioengineering solutions.

## Conclusions

Proper adaptation of species selection in mix-cultures could act as a preventive mechanism of slope failures and reduce the risk of landslides. This soil bioengineering approach is wholesome in the sense that it offers a multitude of benefits right from assisting in ecological restoration, soil rehabilitation to increase in slope stability. Since most highways are constructed by cutting through slopes, the use of plants provides a cost-effective solution as the cost of maintenance could be reduced tremendously once the process of succession takes place to receive a high influx of new species into the community as each plant species comes with a different rooting architecture and different root function to combat slope instability. However, the application of bioengineering approach is limited to the mitigation of low to moderate risk of slope failures and does not extend far beyond that, such as high-risk slopes as the latter favors the use of traditional engineering methods. Nevertheless, a more holistic approach should be utilized to explore and study the interaction between plant, soil properties, ecosystem and the environment.

## Supplemental Information

10.7717/peerj.10477/supp-1 Supplemental Information 1 Root system based on the tap, lateral and horizontal roots (Yen, 1987). (A) H-type (B) R-type (C) VH-type (D) V-type (E) M-type

10.7717/peerj.10477/supp-2 Supplemental Information 2 Root systems and root architecture of selected slope plant species (Saifuddin & Normaniza, 2016).

10.7717/peerj.10477/supp-3 Supplemental Information 3 Suggested planting positions of slope plants based on their rooting characteristics (Saifuddin & Normaniza, 2016).

## Additional Information and Declarations

### Data Availability

The following information was supplied regarding data availability: This is a literature review; there are no raw data.
