# Supplementary figures and images for: Present practices and emerging opportunities in bioengineering for slope stabilization in Malaysia: An overview
Source: PeerJ. 2021 Jan 12;9:e10477. doi: 10.7717/peerj.10477 (PMC7810040; doi:10.7717/peerj.10477)

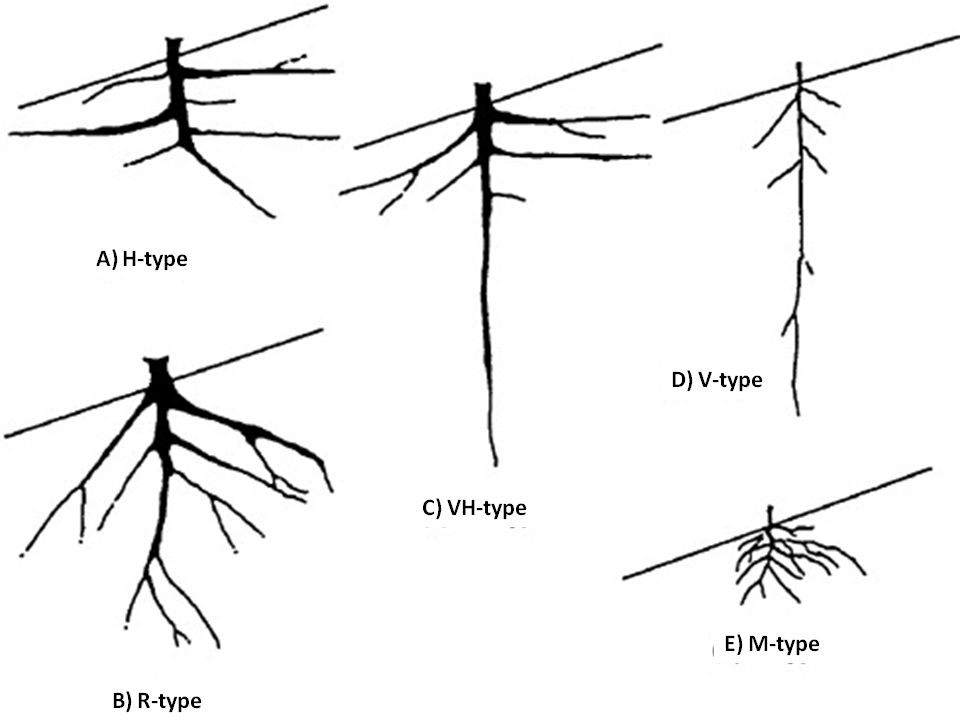

Supplement: Supplemental Information 1 — (A) H-type (B) R-type (C) VH-type (D) V-type (E) M-type [file peerj-09-10477-s001.png]

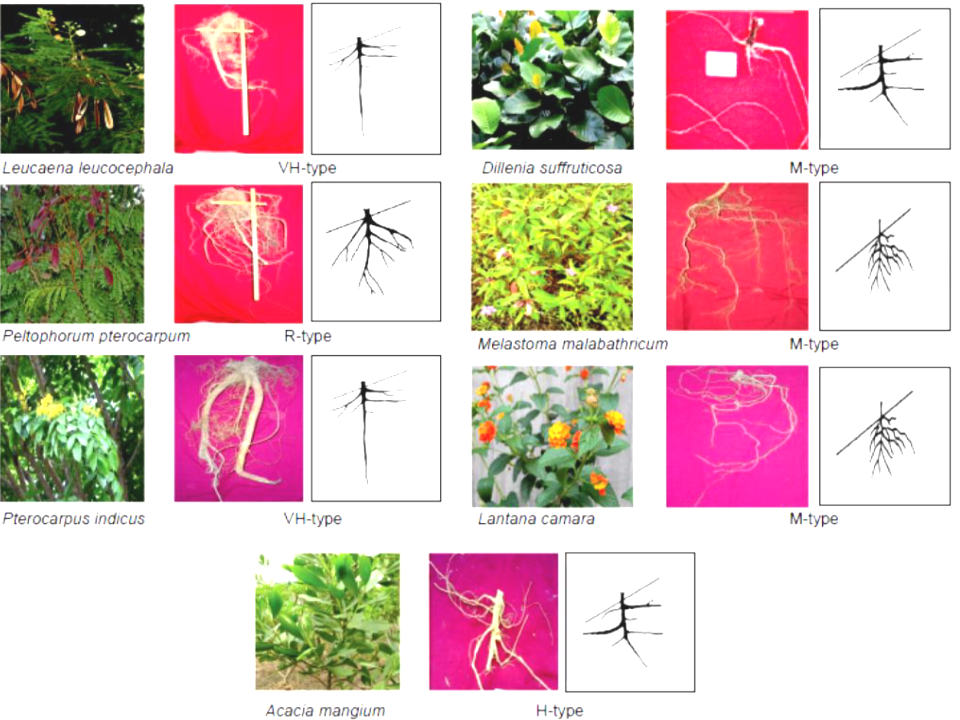

Supplement: Supplemental Information 2 [file peerj-09-10477-s002.png]

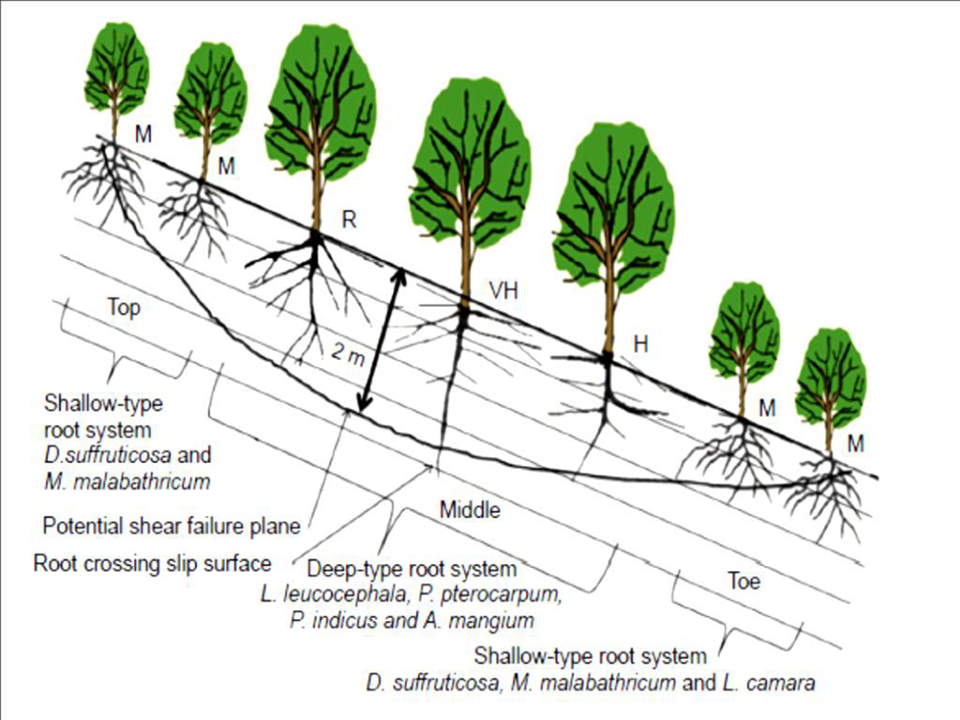

Supplement: Supplemental Information 3 [file peerj-09-10477-s003.png]
